# Evidence of association with type 1 diabetes in the SLC11A1 gene region

**DOI:** 10.1186/1471-2350-12-59

**Published:** 2011-04-27

**Authors:** Jennie HM Yang, Kate Downes, Joanna MM Howson, Sarah Nutland, Helen E Stevens, Neil M Walker, John A Todd

**Affiliations:** 1 Juvenile Diabetes Research Foundation/Wellcome Trust Diabetes and Inflammation Laboratory, Cambridge Institute for Medical Research, University of Cambridge, Addenbrooke's Hospital, Hills Road, Cambridge, CB2 0XY UK

## Abstract

**Background:**

Linkage and congenic strain analyses using the nonobese diabetic (NOD) mouse as a model for human type 1 autoimmune diabetes (T1D) have identified several NOD mouse *Idd *(insulin dependent diabetes) loci, including *Slc11a1 *(formerly known as *Nramp1*). Genetic variants in the orthologous region encompassing *SLC11A1 *in human chromosome 2q35 have been reported to be associated with various immune-related diseases including T1D. Here, we have conducted association analysis of this candidate gene region, and then investigated potential correlations between the most T1D-associated variant and RNA expression of the SLC11A1 gene and its splice isoform.

**Methods:**

Nine SNPs (rs2276631, rs2279015, rs1809231, rs1059823, rs17235409 (D543N), rs17235416 (3'UTR), rs3731865 (INT4), rs7573065 (-237 C→T) and rs4674297) were genotyped using TaqMan genotyping assays and the polymorphic promoter microsatellite (GT)n was genotyped using PCR and fragment length analysis. A maximum of 8,863 T1D British cases and 10,841 British controls, all of white European descent, were used to test association using logistic regression. A maximum of 5,696 T1D families were also tested for association using the transmission/disequilibrium test (TDT). We considered *P *≤ 0.005 as evidence of association given that we tested nine variants in total. Upon identification of the most T1D-associated variant, we investigated the correlation between its genotype and *SLC11A1 *expression overall or with splice isoform ratio using 42 PAXgene whole blood samples from healthy donors by quantitative PCR (qPCR).

**Results:**

Using the case-control collection, rs3731865 (INT4) was identified to be the variant most associated with T1D (*P *= 1.55 × 10^-6^). There was also some evidence of association at rs4674297 (*P *= 1.57 × 10^-4^). No evidence of disease association was obtained at any of the loci using the family collections (*P*_TDT _≥ 0.13). We also did not observe a correlation between rs3731865 genotypes and *SLC11A1 *expression overall or with splice isoform expression.

**Conclusion:**

We conclude that rs3731685 (INT4) in the SLC11A1 gene may be associated with T1D susceptibility in the European ancestry population studied. We did not observe a difference in *SLC11A1 *expression at the RNA level based on the genotypes of rs3731865 in whole blood samples. However, a potential correlation cannot be ruled out in purified cell subsets especially monocytes or macrophages.

## Background

Type 1 diabetes (T1D) is a heritable polygenic autoimmune disease in which both genetic and environmental factors contribute to pathogenesis. To date over 50 loci have been identified that affect risk of T1D [1-3]. The causal genes, variants and haplotypes involved within many of these regions have yet to be identified.

The nonobese diabetic (NOD) mouse develops autoimmune insulin-dependent diabetes spontaneously, which resembles human T1D, and has been widely used to study and map non-MHC T1D loci. The loci identified in the NOD mouse as high priority candidates for human T1D studies include solute carrier family 11 member 1 (*Slc11a1*), which was formerly known as natural resistance-associated macrophage protein 1 (*Nramp1*) [4]. *Slc11a1 *is encoded in the *Idd5.2 *region on mouse chromosome 1. The NOD strain with the disease-predisposing allele expresses the functional protein, whereas the T1D-resistant B10 strain does not [5,6]. The NOD allele was originally identified as providing resistance to bacterial infections in mice. Kissler *et al. *used RNA interference to reduce *Slc11a1 *expression *in vivo *in NOD mice, and found that this reduced the frequency of T1D, mimicking the protective *Idd5.2 *T1D-resistant haplotype [7]. Furthermore, Slc11a1 was found to augment activation of a diabetogenic T-cell clone by enhancing the processing and presentation of pancreatic islet antigens, such as glutamic acid decarboxylase GAD_65_, in dendritic cells (DCs) [8].

In humans, *SLC11A1 *is 14 kb in length with 15 exons. The gene is located in an approximately 400 kb region of high linkage disequilibrium (LD) on chromosome 2q35. The human and mouse SLC11A1 protein sequences have a high degree of conservation, with 88% identity and 93% overall sequence similarity [9]. *SLC11A1 *is expressed in monocytes [10,11], which are the circulating precursors of macrophages and DCs, the major antigen-presenting cells in the immune system. SLC11A1 has pleiotropic effects on macrophage function, all of which are important in resistance to intracellular pathogens. These include release of nitric oxide, L-arginine flux, oxidative burst, tumouricidal and antimicrobial activities, as well as upregulation of CXC chemokine KC, tumour necrosis factor-α, interleukin-1β, inducible nitric oxide synthase and MHC class II expression [12,13]. Roles of monocytes and macrophages have been implicated in the pathogenesis of T1D [14,15]. Recently, macrophages have been shown to be one of the major immune cell populations in infiltrated pancreatic islets of autopsy tissues from patients with T1D [15,16], suggesting that macrophages may contribute to the early phase of beta-cell destruction. Together with the evidence showing that over-activation of SLC11A1 could potentially induce and maintain autoimmune diseases, these data make *SLC11A1 *a candidate gene for autoimmune and immune-mediated disorders, such as T1D. Interestingly, SLC11A1 has been shown to suppress IL-10 production [17], and the gene that encodes IL-10 has recently been associated with T1D susceptibility [1,18], as well as with risk of ulcerative colitis [19] and of systemic lupus erythematosus [20].

Genetic variants in the SLC11A1 gene region have been reported to be associated with various infectious [21-29] and chronic immune diseases, such as T1D [30-33], rheumatoid arthritis (RA) [34-37], juvenile RA (also known as juvenile idiopathic arthritis; JIA) [38,39], sarcoidosis [40], inflammatory bowel disease (IBD) [41-45], Kawasaki disease [46] and multiple sclerosis [47]. More recently, *SLC11A1 *has also been claimed to be associated with Behcet's syndrome in a Turkish population [48] and esophageal cancer in a South African population [49]. Blackwell *et al. *identified a potentially functional polymorphic microsatellite with Z-DNA forming dinucleotide repeats in the promoter region of the human SLC11A1 gene [50,51]. Allele 3 with the apparent stronger promoter activity that drives higher expression of *SLC11A1 *relative to allele 2 may result in chronic macrophage hyperactivation, thus predisposing to autoimmune diseases, but protecting against infectious diseases (see additional file 1 for microsatellite allele sequences) [51]. However, not all studies have replicated the association of the promoter polymorphism in T1D [31,32]. Indeed, associations at other polymorphisms, such as rs17235409 (D543N), rs17235416 (3'UTR) and rs3731865 (INT4), have been reported in immune-related and infectious diseases [22,23,25-28,35-37,39,43], implying that alternative variants may be causal. In IBD, Zaahl *et al. *have shown that the *SLC11A1 *association involves a protective effect of the promoter SNP, rs7573065 (-237 C→T) [44]. They previously found that in the presence of allele 3 of the 5' microsatellite, the change from allele C to T at rs7573065 (-237 C→T) lowered the expression of *SLC11A1*, to a similar level as observed with allele 2 of the microsatellite [52]. Combined, these data suggest that both rs7573065 (-237 C→T) and the microsatellite (r^2 ^= 0.02 and D' = 0.98 in controls; see additional file 2) together might be associated with disease.

Previously, Maier *et al. *found no evidence of association of *SLC11A1 *with T1D using four tag SNPs (rs2276631, rs2279015, rs1059823 and rs1809231), but only 1,709 T1D cases, 1,829 controls and 1,632 families were studied [4]. They also genotyped the non-synonymous SNP (nsSNP) rs17235409 (D543N) and the microsatellite (GT)n in 1,632 families and did not obtain any evidence of association [4]. Nor did they obtain evidence of association with the nsSNP rs17235409 (D543N), which was genotyped in an additional 1,995 cases and 2,101 controls [4]. The genome-wide association study (GWAS) performed by the Wellcome Trust Case Control Consortium (WTCCC) identified a SNP, rs4674297, in *MGC50811 *(also known as *C2orf62*), located within the same LD block as *SLC11A1 *that showed some evidence of association with T1D (*P *= 0.0070) [53]. Barrett and colleagues performed a meta-analysis of three GWAS totalling 7,514 cases and 9,045 controls [1]. They found rs4674297 was one of the four most T1D-associated SNPs in the region with *P*-values of around 10^-4 ^[1]. Following the additional functional support obtained using the NOD mouse model [7,8], we have performed a comprehensive association analysis of sequence polymorphisms in the *SLC11A1 *region in a maximum of 8,863 unrelated T1D cases and 10,841 controls as well as up to 5,696 T1D families in order to identify the most associated, potentially causal, T1D variant(s) and its effect on expression and splicing of the SLC11A1 gene.

## Results and discussion

**Figure 1:**
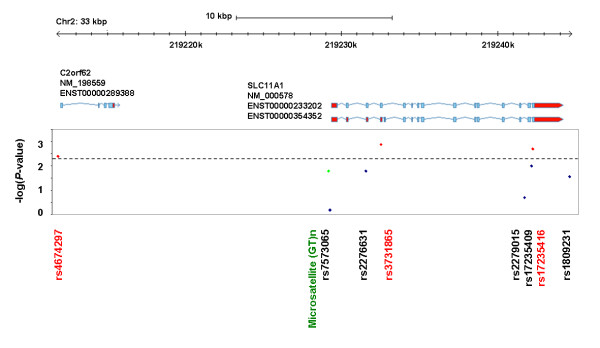
**A schematic of the SLC11A1 gene region with the T1D genetic association results using a maximum of 5,878 cases and 6,406 controls for the SNPs and 7,700 cases and 7,380 controls for the microsatellite (GT)n**. SNPs with *P *≤ 0.005 (horizontal dashed line) were chosen to be genotyped in our extended case-control collection (maximum of 8,863 T1D cases and 10,841 controls) and are shown in red. The microsatellite (GT)n is depicted in green. The multiplicative allelic effects model was an appropriate model for all variants (*P *> 0.05), except for rs1809231 C > G as there was a significant difference between the genotype and the multiplicative allelic effects models (*P *= 0.019; Table 1). [NCBI build 37 was used]

We genotyped and analysed three of the Maier *et al. *tag SNPs (rs2276631, rs2279015 and rs1809231 [4]), the nsSNP rs17235409 (D543N), the indel rs17235416 (3'UTR), rs3731865 (INT4), rs7573065 (-237 C→T) and rs4674297 in up to 5,878 cases and 6,406 controls (Figure 1 and Table 1). As the promoter microsatellite (GT)n has been suggested to be a functional variant, we genotyped this polymorphism in the maximum number of samples in the collection available at the time, which was 7,894 cases and 7,560 controls (Figure 1 and Table 1). Since rs3731865 (INT4), the indel rs17235416 (3'UTR) and rs4674297 showed the most evidence of association with T1D (*P *≤ 0.005; Figure 1), they were genotyped in our extended case-control collection in an additional 2,985 cases and 4,435 controls to test whether their effects were independent.

**Table 1:** Summary of T1D association results for the case-control collection

				Allele or genotype frequency N (%)				
Variant [Synonym] Location	Number of cases	Number of controls	Allele or genotype	in cases	in controls	OR	(95% C.I.)	*P*-value
rs4674297 G > A	8502	10071	A	3710 (21.82)	4682 (23.25)	0.91	(0.86-0.96)	1.57 × 10^-4^
5' of *SLC11A1*								
within *MGC50811 (aka C2orf62)*			G/G	5210 (61.28)	5945 (59.03)	1.00	(reference)	
			G/A	2874 (33.80)	3570 (35.45)	0.91	(0.86-0.97)	
*P*_HWE _= 0.050			A/A	418 (4.92)	556 (5.52)	0.84	(0.74-0.96)	

Microsatellite (GT)n 3 > 2	7697	7371	2	3999 (25.98)	4010 (27.20)	0.94	(0.89-0.99)	0.016
Promoter								
			3*/3*	4195 (54.50)	3913 (53.09)	1.00	(reference)	
			3*/2	3005 (39.04)	2906 (39.42)	0.96	(0.89-1.03)	
*P*_HWE _= 0.697			2/2	497 (6.46)	552 (7.49)	0.85	(0.74-0.97)	

rs7573065 C > T	5649	6233	T	647 (5.73)	732 (5.87)	0.97	(0.87-1.09)	0.662
[-237 C->T]								
Promoter			C/C	5018 (88.83)	5518 (88.53)	1.00	(reference)	
			C/T	615 (10.89)	698 (11.20)	0.97	(0.86-1.09)	
*P*_HWE _= 0.303			T/T	16 (0.28)	17 (0.27)	1.00	(0.50-2.00)	

rs2276631 C > T	5578	6048	T	2933 (26.29)	3331 (27.54)	0.93	(0.87-0.99)	0.016
[274 [C/T]]								
Exon 3			C/C	2998 (53.75)	3153 (52.13)	1.00	(reference)	
			C/T	2227 (39.92)	2459 (40.66)	0.94	(0.87-1.02)	
*P*_HWE _= 0.144			T/T	353 (6.33)	436 (7.21)	0.84	(0.72-0.98)	

rs3731865 G > C	8787	10611	C	4691 (26.69)	6116 (28.82)	0.90	(0.86-0.94)	1.55 × 10^-6^
[469 +14 [G/C]; INT4]								
Intron 4			G/G	4713 (53.64)	5401 (50.90)	1.00	(reference)	
			G/C	3457 (39.34)	4304 (40.56)	0.91	(0.86-0.97)	
*P*_HWE _= 0.242			C/C	617 (7.02)	906 (8.54)	0.78	(0.69-0.87)	

rs2279015 G > A	5549	5872	A	4312 (38.85)	4658 (39.66)	0.96	(0.91-1.02)	0.205
[1465-85 [A/G]]								
Intron 13			G/G	2060 (37.12)	2142 (36.48)	1.00	(reference)	
			G/A	2666 (48.04)	2802 (47.72)	1.00	(0.92-1.08)	
*P*_HWE _= 0.817			A/A	823 (14.83)	928 (15.80)	0.92	(0.82-1.03)	

rs17235409 G > A	5498	6062	A	241 (2.19)	216 (1.78)	1.28	(1.06-1.55)	0.010
[D543N]								
Exon 15			G/G	5259 (95.65)	5849 (96.49)	1.00	(reference)	
			G/A	237 (4.31)	210 (3.46)	1.31	(1.08-1.59)	
*P*_HWE _= 0.430			A/A	2 (0.04)	3 (0.05)	0.72	(0.12-4.37)	

rs17235416 TGTG> del	8463	9835	del	312 (1.84)	299 (1.52)	1.22	(1.04-1.44)	0.015
[1729+55del4 [TGTG]; 3'UTR]								
3'UTR			TGTG/TGTG	8153 (96.34)	9539 (96.99)	1.00	(reference)	
			TGTG/del	308 (3.64)	293 (2.98)	1.24	(1.05-1.46)	
*P*_HWE _= 0.624			del/del	2 (0.02)	3 (0.03)	0.69	(0.11-4.20)	

rs1059823 A > G	5,605	6,137	ND	ND	ND	ND		ND
[1801+86[A/G]]								
3'UTR								
*P*_HWE _= 0.001								

rs1809231 C > G	5643	6151	G	4870 (43.15)	5399 (43.89)	0.97	(0.92-1.02)	0.028**
3' intergenic region								
			C/C	1783 (31.60)	1959 (31.85)	1.00	(reference)	
			C/G	2850 (50.51)	2985 (48.53)	1.04	(0.96-1.14)	
*P*_HWE _= 0.249			G/G	1010 (17.90)	1207 (19.62)	0.91	(0.82-1.01)	

Association of alleles and genotypes were tested using single locus logistic regression.

* Rare microsatellite alleles (with frequency <0.1%) were combined with the common allele 3. ** The multiplicative allelic effects model was an appropriate model for all variants (*P *> 0.05), except for rs1809231 C>G where a genotype effects model was required (*P *= 0.019). [*P*_HWE _= Hardy- Weinberg equilibrium in controls; OR = odds ratio; 95% C.I. = 95% confidence intervals; ND = not done.]

We had over 96% power to detect an effect size of 0.90, at an alpha level of 0.005, assuming a multiplicative allelic effects model and a minor allele frequency (MAF) of 0.29, with a sample size of 8,863 cases and 10,841 controls. rs3731865 (INT4) showed the most evidence of association with T1D (*P *= 1.55 × 10^-6^; OR = 0.90 (95% confidence interval (C.I.) 0.86-0.94); Table 1). The support for association with T1D at rs4674297, a SNP identified from the Barrett *et al. *meta-analysis study (*P *= 2.9 × 10^-4^) [1], was maintained (*P *= 1.57 × 10^-4^; OR = 0.91 (95% C.I. 0.86-0.96); Table 1; 5,897 cases and 5,461 controls in our full dataset of 8,863 cases and 10,841 controls overlapped with 7,514 cases and 9,045 controls in the Barrett *et al. *meta-analysis study). Our samples were not genotyped at the other three SNPs, rs12471773, rs2290708 and rs3816560, found in the meta-analysis as they are in high LD with the associated SNPs (rs3731865 and rs4674297; r^2 ^> 0.8 in controls) and so are unlikely to significantly improve the T1D association signal in this region. We found no evidence of associations that were independent of rs3731865 (INT4) (*P *> 0.007).

We obtained no evidence of association with T1D at any of the nine *SLC11A1 *polymorphisms genotyped with T1D in up to 5,696 families (*P *≥ 0.13; Table 2). For the most T1D-associated SNP, rs3731865 (INT4), the TDT result indicated no association (RR = 1.00 (0.95-1.06), *P *= 0.98; Table 2) despite being genotyped in the largest number of families. This could have been due to a modest level of power (53%) in the family collections to detect an effect size of RR = 0.90 with MAF of 0.26 at an alpha level of 0.005, assuming a multiplicative allelic effects model. This study had 82% power at an alpha level of 0.05.

**Table 2:** Summary of T1D association results for the family collections

Variant	Number of families	Number of informative transmissions	Minor allele frequency (%) in affected siblings	Minor allele frequency (%) in unaffected parents	Relative risk (95% C.I.)	Family ***P***_**TDT**_
rs4674297 G>A	NA	NA	NA	NA	NA	NA
Microsatellite (GT)n 3*>2	1971	1925	27.02	27.06	1.00 (0.92-1.10)	0.87
rs7573065 C>T	2472	610	5.43	5.30	0.92 (0.78-1.08)	0.29
rs2276631 C>T	2707	2587	27.38	27.15	1.00 (0.93-1.08)	0.98
rs3731865 G>C	5010	4876	26.43	26.42	1.00 (0.95-1.06)	0.98
rs2279015 G>A	2523	2862	36.33	36.26	0.94 (0.88-1.02)	0.13
rs17235409 G>A	1859	198	2.05	2.10	0.92 (0.70-1.22)	0.57
rs17235416 TGTG>del	2450	191	1.61	1.65	0.85 (0.64-1.14)	0.28
rs1059823 A>G	2591	2950	39.79	38.81	1.02 (0.95-1.10)	0.56
rs1809231 C>G	2678	3166	42.71	42.14	1.01 (0.94-1.08)	0.78

Number of families with genotyping data for the variants in the SLC11A1 gene region with the respective number of informative transmissions, the relative risk and the *P*-value from the transmission/disequilibrium test (*P*_TDT_). [* Rare microsatellite alleles, with frequency <0.1%, were combined with the common allele 3; 95% C.I. = 95% confidence interval; NA = not available]

The 2004 IBD study by Zaahl *et al. *suggested that the promoter SNP, rs7573065 (-237 C→T), and the microsatellite (GT)n might, in combination, be associated with disease [52]. However, these variants are not associated with T1D, either together in a joint effects model (*P *= 0.33) or individually (*P *> 0.01; Table 1). We also assessed whether rs7573065 (-237 C→T) and the microsatellite were involved with T1D susceptibility in a haplotypic manner. Haplotype 3.T (microsatellite.rs7573065) was grouped with all the other haplotypes consisting of the protective allele 2 of the microsatellite, since they were found to exert similar effects on transcriptional activity [52]. Using the most 'susceptible haplotype', 3.C, as the reference, the grouped haplotypes did not show an association with T1D (*P *= 0.15; OR = 0.96 (95% C.I. 0.90-1.02)). Furthermore, haplotype 3.T by itself did not confer protection against T1D (*P *= 0.32; OR = 0.94 (95% C.I. 0.83-1.07)). Together these analyses suggest that rs7573065 (-237 C→T) does not confer protection against T1D, either by itself or in combination with allele 3 of the microsatellite.

*SLC11A1 *has several known alternative splice transcripts of which the two major isoforms expressed are full length transcripts with or without exon 4a (74 bp in length). Previous studies have shown that exon 4a, which is located between exon 4 and 5, encoded by an Alu element, is transcribed *in vivo *but would introduce two termination codons in exon 5 resulting in severely truncated, and thus non-functional, SLC11A1 protein [9]. Interestingly, the most T1D-associated *SLC11A1 *polymorphism, rs3731865, is located in intron 4, 13 bp 3' of exon 4 and 167 bp 5' of the alternatively spliced exon 4a. Although bioinformatics analyses failed to predict known splice elements around or at rs3731865, due to its location within the gene, we hypothesised that rs3731865 might affect elements for transcription or splicing of *SLC11A1 *exons. This splicing could cause changes in functional message levels and the amount of functional SLC11A1 protein expressed. Therefore, measuring the ratio of transcripts with or without exon 4a in the cell population of interest and correlating the expression of *SLC11A1 *with genotypes of rs3731865 could be informative for determining the effect of this SNP on gene expression.

**Figure 2:**
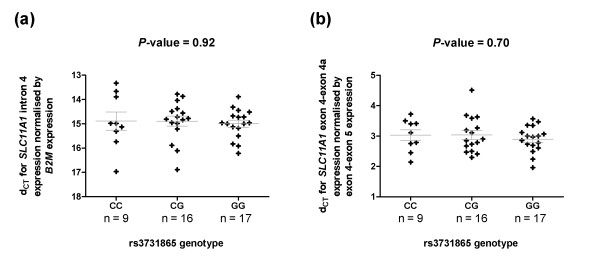
***SLC11A1 *expression by rs3731865 genotype**. No genotype effect was detected either for (a) overall expression or for (b) the ratio of both isoforms. [C = minor protective allele and G = major susceptibility allele]

*SLC11A1 *mRNA is expressed specifically in whole blood, CD14^+ ^monocytes, CD33^+ ^and CD66b (granulocytes) myeloid cells according to the microarray expression data available from BioGPS and HaemAtlas [10,11]. Since whole blood samples were available in the laboratory, we investigated if the genotypes of rs3731865 correlated with the overall expression of *SLC11A1 *and also with the isoform ratio in whole blood samples collected in PAXgene RNA tubes from 42 healthy donors recruited from Cambridge BioResource (CBR) [54]. It was hypothesised that in donors homozygous for the T1D susceptibility allele, there would be increased levels of transcripts without exon 4a, which codes for functional protein, and less of the alternative transcript, which encodes premature stop codons and, thus, a non-functional protein. Thereby an overall higher splice isoform ratio is expected in individuals homozygous for the susceptibility allele compared to individuals homozygous for the protective allele. However, no genotype effect was detected for expression of *SLC11A1 *overall or for isoform ratio differences (*P *= 0.92 and 0.70, respectively; Figure 2).

The failure to detect *SLC11A1 *expression differences based on rs3731865 genotypes could be due to various reasons, such as there is actually no correlation between its transcriptional splicing and genotypes of rs3731865 in whole blood, or there is, but the quantitative PCR (qPCR) assays are not sensitive enough to detect it, or perhaps the current sample size may not be large enough to have sufficient power to detect a small difference. It is also possible that a purified cell subset should be studied instead of analysing overall expression in whole blood, such as monocyte-derived macrophages, where the gene is known to function.

The incidence of T1D is predicted to double in children under 5 years by 2020 [55], with the likely cause being the changing environment. These environmental changes could impact our association study, with gene-environment interactions becoming increasingly important. Our control cohort is of similar age to the parents of our cases. This single generational difference could still reduce our power to find effects in our case-control study in the presence of gene-environment interactions, owing to their differences in early life environmental exposures. However, this does not affect the significance of the findings obtained. Taking the data collectively, we identified the strongest allelic association with T1D at rs3731865 in intron 4 of *SLC11A1*. This SNP is also most associated with JIA in Finnish families in the same direction where the minor allele is also protective for disease [39]. Although the promoter microsatellite remains the only variant reported to date for which there is evidence of a correlation with function in the SLC11A1 gene region [43,51], alleles 2 and 3 of the microsatellite are in strong LD with rs3731865 (r^2 ^= 0.79 in controls; see additional file 2). Therefore, any T1D association detected for the microsatellite might be tagging the association of rs3731865. The extensive prior evidence linking SLC11A1 function and expression with autoimmune, immune-related and infectious diseases in mice and humans, and the confirmed polygenic model of T1D inheritance of numerous small effects, suggest that *SLC11A1 *variation may well have a very small effect on human T1D risk [56,57].

A search for additional rare variants via next generation sequencing, as well as results coming from the 1000 Genomes Project [58], could provide further information regarding the possibility of an association of *SLC11A1 *with T1D. Recently, Zeller *et al. *and Heinig *et al. *performed a large-scale investigation of the transcriptome of circulating monocytes and monocyte-differentiated macrophages using the Illumina Human HT-12 expression BeadChips [14,59]. The two microarray probes (ILMN_1741165 and ILMN_1735737) in *SLC11A1 *mapped to the 3'UTR of the gene. Using the data from Heinig *et al. *a stronger expression signal is detected by the ILMN_1741165 probe compared to the other probe [14]. Although expression quantitative trait loci (eQTL) and eSNPs for *SLC11A1 *were identified from these studies for the ILMN_1741165 probe, including the T1D-associated SNP, rs4674297 (*P *= 1.03 × 10^-65^) [59], this probe maps to repeat sequences [14,59]. Therefore the eQTL identified for *SLC11A1 *is highly questionable and could be an artefact. Only about 39% of eQTLs are likely to be due to "simple effect" SNPs, those that altered transcription levels [60]. The majority of eQTL variations are, however, owed to other alterations in gene expression, namely altered initiation/termination and/or splicing [60]. Hence, analysis of microarrays with multiple probes per gene and per exon *e.g. *Affymetrix Gene Array, and, in the future, direct RNA sequencing using the next generation technologies should be more informative. Investigating the correlation of *SLC11A1 *RNA expression and protein level in genotyped human macrophage/stimulated monocyte or DC samples based on the genotype/haplotype of the most T1D-associated variant(s) would help to prove causality of the genes in human T1D and help to identify biological disease mechanisms involved in pathogenesis of T1D.

## Conclusion

We conclude that genetic variation of the SLC11A1 gene could possibly be associated with T1D susceptibility. We did not observe a difference in *SLC11A1 *expression at the RNA level based on the genotypes of the SNP most associated with T1D, rs3731865 (INT4), in whole blood samples. However, a potential correlation cannot be ruled out in purified blood cell subsets.

## Methods

### Subjects

We genotyped a maximum of 8,863 T1D cases and 10,841 controls of self-reported white ethnicity collected from across Great Britain, with non-European subjects excluded by GWAS genotyping results (see Genetic association statistical analysis). All cases were recruited as part of the Juvenile Diabetes Research Foundation/Wellcome Trust Diabetes and Inflammation Laboratory U.K. Genetic Resource Investigating Diabetes (GRID) study [61]. All cases were diabetic defined using the WHO criteria. Most were less than 16 years of age at the time of collection and all were diagnosed before 17 years of age, with a mean age-at-diagnosis of 7.8 years. Controls were obtained from the British 1958 Birth Cohort (n = 7,658 [62]) and the UK Blood Service's collaboration with the WTCCC (n = 3,183 [63]). A collection of 5,696 families of European ancestry were also genotyped. These included 470 multiplex families from the Diabetes UK Warren 1 repository, 263 simplex families from Northern Ireland, 80 simplex families from Yorkshire, 734 simplex families from Finland, 360 simplex families from Norway, 423 simplex families from Romania, 335 multiplex families from the Human Biological Data Interchange (U.S.) and 3,031 families made available through the T1DGC [64]. The T1DGC families comprised of 362 families from the Asia-Pacific region, 873 families from across Europe, 1,633 from North America and 163 from the U.K. [65]. All families had samples from both parents and at least one affected child. Families exhibiting misinheritances were excluded. All DNA samples were collected with approval from the relevant research ethics committees and written informed consent was obtained from the participants or their guardians, if they were too young to consent.

PAXgene whole blood samples were obtained from 42 healthy subjects of self-reported white ethnicity with non-autoimmune disease status from the Cambridge BioResource (CBR) [54] and selected by *SLC11A1 *genotype. T1D patients were excluded in this study to avoid any confounding due to T1D status or to insulin treatment. Informed consent was obtained from all volunteers upon recruitment to the CBR for the collection and use of DNA samples for genotyping. Ethical approval for this study was given by the Cambridgeshire 3 Research Ethics Committee and an informed consent was obtained for the collection and use of the fresh peripheral blood samples.

### Genotyping

Nine SNPs (rs2276631, rs2279015, rs1809231, rs1059823, rs17235409, rs17235416, rs3731865, rs7573065 and rs4674297) were genotyped using Custom TaqMan^® ^SNP Genotyping Assays (Applied Biosystems (ABI)) according to manufacturers' protocols. The microsatellite (GT)n was amplified using AmpliTaq^® ^(ABI) using 5' FAM labelled forward primer (5'-6ttcaatgcatgtcccttctg-3') and reverse primer (5'-ccatggagtggacctttgtt-3') in 6 μl reaction volume with the following PCR conditions: an initial denaturation at 95°C for 5 minutes, then 25 cycles of denaturation at 94°C for 30 seconds, annealing at 65°C for 30 seconds and extension at 72°C for 30 seconds, with a final extension at 72°C for 10 minutes. 2 μl of PCR amplified product was added to 10 μl of pre-diluted GeneScan LIZ500 size standard (25 μl of LIZ500 in 1 ml of Hi-Di Formamide; ABI) and heated at 95°C for 5 minutes before being resolved on ABI PRISM 3730XL Genetic Analyzer (ABI) and scored on GeneMapper v.4.0 software (ABI).

Case and control DNA samples were distributed evenly on 384-well plates for genotyping. All genotyping data were scored by one researcher and reviewed by a second to minimise error. Both researchers were unaware of case-control status and family structure. 58 out of 2,275 96-well case-control DNA plates (2.5%) in this experiment had some scoring differences, 29 in one well only, and with only one DNA plate (of whole genome-amplified samples) requiring substantial review. Genotyping success rates ranged from 93.3% to 97.5% for the nine SNPs and the microsatellite (GT)n. Across the full case-control collection, there were a maximum of 298 duplicates, the majority (262) being control samples that have been submitted twice. There were no duplicates in the family collections. Across the nine SNPs and the microsatellite the overall concordance rates were 99.6% and 96.9%, respectively. Hardy-Weinberg equilibrium was calculated on a plate-by-plate basis in addition to the entire case and control samples to highlight any erroneous plates. Genotypes of rs1059823 were found to deviate significantly from Hardy-Weinberg equilibrium in the controls (*P *= 0.001) and, therefore, rs1059823 was not tested for association with T1D using the case-control collection.

### Genetic association statistical analysis

All statistical analyses were performed in the statistical package, STATA v10.0 [66]. There was no difference in allele frequencies between the two control cohorts [53]. Most of the samples used here have also been part of a GWAS and as such have been subjected to rigorous testing for population heterogeneity [1,53]. Any non-Caucasian and related individuals identified through previous GWAS have also been removed [1,53,56,67,68]. Association with T1D was tested using logistic regression models. Cases and controls were matched according to 12 broad geographical subregions of England, Scotland and Wales (Southwestern, Southern, Southeastern, London, Eastern, Wales, Midlands, North Midlands, Northwestern, East and West Riding, Northern, and Scotland), based on the place of recruitment and the place of birth, respectively, to minimise confounding due to geographical differences in allele frequency and disease incidence. Each genetic variant was first tested for association using a multiplicative allelic effects model on one degree of freedom (df), with counts of the minor allele, coded 0, 1, 2, as the dependent variable, disease status as the outcome variable and geographical subregions included as strata. Each variant was tested using a genotype effects model (on two df) that did not assume a specific inheritance model. The genotype and allelic effects models were compared using a likelihood ratio test. Except for rs1809231, the multiplicative allelic effects model was reported as it was not significantly different from the genotype effects model (*P *> 0.05).

Although the reported *P*-values were not adjusted for multiple testing, the *P*-value threshold required for statistical significance was adjusted. We performed nine independent tests and so only considered an association significant if *P *≤ 0.005. Power calculations for the genetic association analyses for the case-control dataset was performed using CaTS as described in Skol *et al. *[69], and for the family datasets, using the method developed by Knapp [70].

Forward logistic regression analyses were performed to test for associations with T1D that were independent of the effect of rs3731865, in other words, evidence against the most significant SNP, rs3731865, being sufficient to model the association in the *SLC11A1 *region [71]. Each variant in turn was added to a model that included rs3731865, and a likelihood ratio test was used to assess whether there was an additional independent effect.

To estimate the joint effects of the microsatellite (GT)n and rs7573065 (-237 C→T SNP), both variants were included in the logistic regression model, under the assumption of multiplicative allelic effects.

Haplotype logistic regression analysis was also carried out for the microsatellite (GT)n and rs7573065 (-237 C→T SNP). Phased haplotypes were generated under the null hypothesis that case and control haplotypes were drawn from the same population using the SNPHAP program, version 1.3.1. 1,000 Expectation Maximum (EM) iterations were used, each started from a random imputation, and only using samples genotyped at both loci. Haplotypes were tested for association in a logistic regression model, where haplotype assignments were weighted by their posterior probabilities, and robust variance estimates were used to account for non-independence when multiple haplotype assignments were possible for each subject.

The transmission/disequilibrium test (TDT) was used to analyse family data for association with disease [72].

### SLC11A1 gene expression assays

Whole blood samples from 42 healthy CBR donors were collected directly into PAXgene Blood RNA tubes (PreAnalytiX). RNA was extracted on the day of blood sample collection using the PAXgene Blood RNA kit (QIAGEN/BD) with DNase I treatment according to manufacturers' protocols. 1 μg of total RNA was used for each reverse transcription (RT) reaction for synthesising cDNA primed with oligo dT primer (18-mer) using Superscript™ III (Invitrogen) according to manufacturers' instructions. To assess whether RNA samples were contaminated with genomic DNA, a corresponding RT-negative control template was set up for each RT reaction. A qPCR assay designed to amplify genomic DNA was used to detect DNA contamination within the RT-negative control sample. *SLC11A1 *qPCR assays were designed using Primer3Plus software [73] and checked for specificity using the Basic Local Alignment Search Tool (BLAST; see additional file 3 for primer and probe sequences). qPCR reactions were run in MicroAmp™ Optical 384-well plates (ABI) in a total reaction volume of 20 μl containing 2 μl of cDNA sample, 0.6 μl of 10 μM forward and reverse primers each, 0.8 μl of 5 μM probe, 10 μl of TaqMan Universal PCR Master Mix (ABI) and 6 μl of dH_2_O. The thermal cycling conditions started with 50°C for 2 minutes, initial denaturation at 95°C for 10 minutes, followed by 40-50 cycles of denaturation at 95°C for 15 seconds and annealing/extension at 60°C for 1 minute. Samples were run in duplicates on the same plate, and fluorescent signals detected using an ABI 7900HT plate reader (ABI) with SDS v2.2.2 software (ABI). Data was analysed using comparative cycle number (C_T_) method, averaged and normalised by the endogenous *B2M *control ran on the same plate. Correlations between *SLC11A1 *overall gene expression and splice isoform ratio with rs3731865 genotypes were tested using ANOVA using the GraphPad Prism software.

## Competing interests

The authors declare that they have no competing interests.

## Authors' contributions

JHMY performed SNP genotyping, statistical analyses, whole blood sample processing, qPCR, collated the data, generated tables and figures and writing the manuscript. KD contributed to whole blood sample processing. JMMH assisted with statistical analysis and helped to draft the manuscript. SN and JAT are members of the CBR Management Committee who had a primary role in the creation and management of the CBR. HES was responsible for the DNA sample management. NMW managed the data. JAT participated in the conception and design of the study, analysed results and helped to draft the manuscript. All authors read and approved the final manuscript.

## Pre-publication history

The pre-publication history for this paper can be accessed here:

http://www.biomedcentral.com/1471-2350/12/59/prepub

## Supplementary Material

Additional file 1 **Sequences of the human *SLC11A1 *polymorphic microsatellite and the allele frequencies in controls**. Click here for file

Additional file 2 **The pair-wise linkage disequilibrium (as measured by r**^**2 **^**and D') between the genotyped variants in the SLC11A1 gene region using the genotyping data from 10,841 controls**. Click here for file

Additional file 3 **Quantitative PCR primer and probe sequences**. Click here for file
