# Supplementary material for: Evidence of association with type 1 diabetes in the SLC11A1 gene region
Source: BMC Med Genet. 2011 Apr 27;12:59. doi: 10.1186/1471-2350-12-59 (PMC3114708; doi:10.1186/1471-2350-12-59)
Supplement: Additional file 1 — Sequences of the human SLC11A1 polymorphic microsatellite and the allele frequencies in controls. [file 1471-2350-12-59-S1.DOC]

**Sequences of the human *SLC11A1* polymorphic microsatellite and the allele frequencies in controls**

| **Allele** | **Sequence** | **PCR product sizes (bp)** | **Frequency in controls (%)** |
| --- | --- | --- | --- |
| 1 | t(gt)_5_ac(gt)_5_ac(gt)_11_ggcaga(g)_6_ | 357 | 0.09 |
| 2 | t(gt)_5_ac(gt)_5_ac(gt)_10_ggcaga(g)_6_ | 355 | 27.20 |
| 3 | t(gt)_5_ac(gt)_5_ac(gt)_9_ggcaga(g)_6_ | 353 | 72.67 |
| 4 | t(gt)_5_ac(gt)_9_ggcaga(g)_6_ | 341 | 0.01 |
| 9 | predicted sequence t(gt)_5_ac(gt)_5_ac(gt)_8_ggcaga(g)_6_ | 351 | 0.02 |
| 349 | predicted sequence t(gt)_5_ac(gt)_5_ac(gt)_7_ggcaga(g)_6_ | 349 | 0.01 |
