# Supplementary figures and images for: Evidence of association with type 1 diabetes in the SLC11A1 gene region
Source: BMC Med Genet. 2011 Apr 27;12:59. doi: 10.1186/1471-2350-12-59 (PMC3114708; doi:10.1186/1471-2350-12-59)

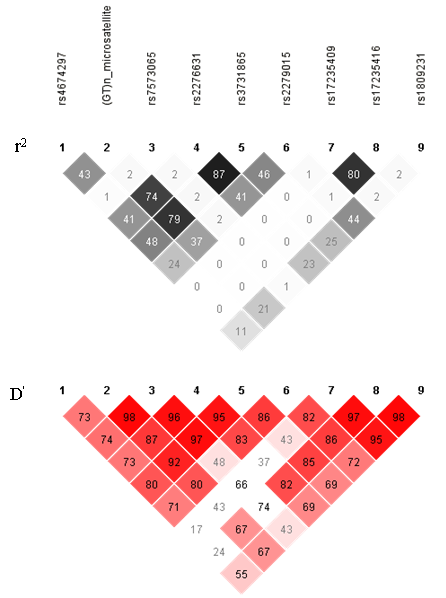

Supplement: Additional file 2 — The pair-wise linkage disequilibrium (as measured by r2 and D') between the genotyped variants in the SLC11A1 gene region using the genotyping data from 10,841 controls. [file 1471-2350-12-59-S2.BMP]
