# Supplementary material for: Evidence of association with type 1 diabetes in the SLC11A1 gene region
Source: BMC Med Genet. 2011 Apr 27;12:59. doi: 10.1186/1471-2350-12-59 (PMC3114708; doi:10.1186/1471-2350-12-59)
Supplement: Additional file 3 — Quantitative PCR primer and probe sequences. [file 1471-2350-12-59-S3.DOC]

### Quantitative PCR primer and probe sequences

| **Assay** | **Forward primer** | **Reverse primer** | **Taqman Probe (5' FAM and 3' TAMRA labelled)** |
| --- | --- | --- | --- |
|  |  |  |  |
| *SLC11A1* exon 4-exon 5 | TCTACTACCCTAAGGTGCCCC | CCAGCTGAGAGCAGATTGAA | AGGAAGTCATCGGCACGGCC |
|  |  |  |  |
| *SLC11A1* exon 4-exon 4a | CTACCCTAAGTCGGAGTCTCG | CCAGCTGAGAGCAGATTGAA | AGGAAGTCATCGGCACGGCC |
|  |  |  |  |
| *SLC11A1* intron 4 | TGGACAGGGAGAACCACTG | GCCCCATGTGAGATGATGTA | CCCCAAACCCCAAACAGCCA |
|  |  |  |  |
| *B2M* | TGCTCGCGCTACTCTCTCT | TCCATTCTCTGCTGGATGAC | CTGGAGGCTATCCAGCGTACTCCAA |
